# Artificial Intelligence Reporting Guidelines’ Adherence in Nephrology for Improved Research and Clinical Outcomes

**DOI:** 10.3390/biomedicines12030606

**Published:** 2024-03-07

**Authors:** Amankeldi A. Salybekov, Markus Wolfien, Waldemar Hahn, Sumi Hidaka, Shuzo Kobayashi

**Affiliations:** 1 Kidney Disease and Transplant Center, Shonan Kamakura General Hospital, Kamakura 247-8533, Japan; s_hidaka@shonankamakura.or.jp (S.H.); shuzo@shonankamakura.or.jp (S.K.); 2 Shonan Research Institute of Innovative Medicine, Shonan Kamakura General Hospital, Kamakura 247-8533, Japan; 3 Qazaq Institute of Innovative Medicine, Astana 010000, Kazakhstan; 4 Carl Gustav Carus Faculty of Medicine, Institute for Medical Informatics and Biometry, Technische Universität Dresden, 01317 Dresden, Germany; waldemar.hahn@tu-dresden.de; 5 Center for Scalable Data Analytics and Artificial Intelligence (ScaDS.AI), 01317 Dresden, Germany

**Keywords:** artificial intelligence, AI reporting guidelines, nephrology, clinical trials, clinical decision support

## Abstract

The use of artificial intelligence (AI) in healthcare is transforming a number of medical fields, including nephrology. The integration of various AI techniques in nephrology facilitates the prediction of the early detection, diagnosis, prognosis, and treatment of kidney disease. Nevertheless, recent reports have demonstrated that the majority of published clinical AI studies lack uniform AI reporting standards, which poses significant challenges in interpreting, replicating, and translating the studies into routine clinical use. In response to these issues, worldwide initiatives have created guidelines for publishing AI-related studies that outline the minimal necessary information that researchers should include. By following standardized reporting frameworks, researchers and clinicians can ensure the reproducibility, reliability, and ethical use of AI models. This will ultimately lead to improved research outcomes, enhanced clinical decision-making, and better patient management. This review article highlights the importance of adhering to AI reporting guidelines in medical research, with a focus on nephrology and urology, and clinical practice for advancing the field and optimizing patient care.

## 1. Introduction

The rapid development of computing technology and the increase in digital data for subsequent analysis has led to an unprecedented increase in research activity in the field of artificial intelligence (AI) and its use in healthcare. Health authorities and medical societies have emphasized the need for predictive models of renal diseases that adapt to routine clinical practice and improve decision-making and patient management. Traditional statistical methods are commonly used in analyzing medical datasets. However, the integrative analyses of heterogeneous medical datasets, which include histological images, time series data in electronic health records, and complex omics data (collectively known as big medical data), have paved the way for novel, advanced AI algorithms to investigate examination findings in a more effective manner [1,2]. Various AI-based studies have demonstrated that the disease diagnostic and prognostic potential of AI tools is promising, especially in histology, to detect cancer tissues (e.g., renal cancer) [3,4,5,6] (Table 1). 

## 2. Methods

Guidelines, studies, and research articles on the keywords “artificial intelligence”, “artificial intelligence in the medical field”, “artificial intelligence guidelines”, and “artificial intelligence in nephrology” were searched in PubMed, Google Scholar, and Scopus databases. Manual searching for the reference lists of eligible studies was performed and only guidelines, studies, and research articles written in English were included. The criteria for exclusion were as follows: (i) non-English written studies; (ii) conference abstracts, notes, letters, case reports, or animal studies; and (iii) duplicate studies.

## 3. Common Ground for AI-Based Clinical Guidelines via FAIR Common Data Models

Since high quality datasets are, overall, still sparse in the medical domain, large scale efforts to collect and anonymously share medical data are in high demand. One prominent development in this direction can be attributed to the OMOP, which stands for Observational Medical Outcomes Partnership [9]. The OMOP was created to develop and promote the use of common data models (CDMs) for observational research in healthcare, thus a CDM is essentially a standardized way of organizing and representing healthcare data from various sources (e.g., electronic health records, claims data, etc.) and multiple sites across institutions, so that it can be used for broader research and analysis applications, including nephrology [6].

One of the key benefits of using the OMOP CDM for cancer research and beyond is that it allows for the more efficient and standardized AI-based analyses of healthcare data [5]. Likewise, this supports computational researchers and clinicians to more easily combine and investigate highly individual patient data, because it can create more diverse and comprehensive datasets. For example, researchers can use an OMOP CDM to conduct studies that examine the effectiveness of different cancer treatments, the factors that contribute to cancer progression or recurrence, and the impact of comorbidities on cancer outcomes [10]. They can also utilize an OMOP CDM to identify patient subgroups that may be at a higher risk for certain types of cancer or that may benefit from specific treatments [11]. In addition, an OMOP CDM can help to support the development and validation of predictive AI models for cancer outcomes [12]. By integrating data from multiple sources into a standardized format, researchers can build and test models that can be used to identify patients who are at a higher risk for cancer or who may benefit from personalized treatment plans. Moreover, the Radiology Common Data Model (R-CDM) for the standardization of Digital Imaging Communications in Medicine (DICOM) was published in 2022 [13]. The R-CDM contains 75,000 radiology terms to harmonize DICOM imaging data into two extended tables, radiology occurrence and radiology image, on the OMOP CDM. This is one of many attempts to combine the high potential of an OMOP CDM containing tabular data and the broadly available sets of medical image data. Besides the OMOP, other CDMs exist that can be utilized for the same tasks, like i2b2 [14], the Patient-Centered Outcomes Research Network (PCORnet) CDM [15] or the CDISC SDTM [16], in which the latter is designed for clinical trials.

In particular, medical image analysis of polycystic kidney disease’s progression already shows essential findings [17,18]. Here, it can be seen that AI is actively penetrating into various fields of medicine, including nephrology and transplant fields. One of the prime examples is that AI-powered donor and recipient data analysis can improve predictions of both short- and long-term graft survival [19,20,21]. These novel algorithms enable the generation of computational models that can learn automatically, generate predictions from prior knowledge and experience on a given topic, and improve information processing without the need to explicitly and manually investigate all possible cases. Moreover, algorithms can often improve their abilities by gaining new experiences that refine and improve the system by providing more knowledge about the problem they are trying to solve, such as image data augmentation [22] or the oversampling of tabular data [23].

However, recent research shows that the majority of AI-powered clinical trials are poorly reported [24,25]. This may raise research concerns about their successful translation and use in clinical settings. In response to these issues, worldwide initiatives have created guidelines for publishing AI-related studies that outline the minimum necessary information that researchers should include.

In addition, research, as well as clinical data are increasing and are collected by adhering to the FAIR data principles, which are a set of guidelines for making data Findable, Accessible, Interoperable, and Reusable (FAIR). These principles are essential for ensuring that data can be used effectively in clinical research and practice [26]. In this light, a CDM should also be designed to be FAIR, as follows: (i) “Findable” by providing a standardized way of organizing and representing healthcare data that can be easily shared and accessed by researchers across different organizations and countries; (ii) “Accessible” by providing open-source tools and documentation to support the use of the CDM in research; (iii) “Interoperable” by providing a common data model that can be used to integrate data from multiple sources and to support standardized analysis and research; and finally (iv) “Reusable” by providing a flexible and adaptable framework that can be used for a wide range of research questions and applications. Taken together, CDMs are designed to support the FAIR principles for scientific data management and stewardship and they have been widely adopted by the research community for their ability to promote open and collaborative research in medicine [27]. In particular, for clinical AI models, adhering to the FAIR principles is of the utmost importance, to allow for a transparent, trustworthy, and reliable development of tools that can help advance clinical research and practice in a responsible and ethical manner [28].

In essence, the main goals of reporting guidelines are to ensure that findings can be understood by readers and reviewers, replicated by other researchers, utilized by healthcare practitioners to make clinical decisions, and included in systematic reviews and meta-analyses [29]. This review article highlights an overview of AI reporting guidelines and their application in healthcare research to support researchers in biomedical domains, including nephrology and transplantation, to improve the overall design, reporting, and, ultimately, the quality of their underlying AI studies.

## 4. What Are AI Clinical Research Reporting Guidelines?

An AI reporting guideline is a brief checklist or structured text using clear methodology for healthcare researchers to support authors in conducting a certain type of research study [29]. In general, a reporting guideline provides a minimum set of information needed to ensure that a manuscript or underlying application can be, for example (i) understood by a broad or more specific readership (e.g., layman or novice readers, domain experts of a related field, domain experts of a different field, computational experts, and/or biomedical experts), (ii) replicated by a researcher of related expertise, (iii) used by a doctor to assist with a clinical decision, and (iv) included in a systematic review or meta-analysis [29]. Reporting guidelines enhance the study design, delivery, and, ultimately, the study quality by providing a concise set of the minimal information that should exist in a document, which can, of course, also be utilized by CDMs [30]. The enhanced completeness and transparency of a research study also contributes to the detection of more visible areas of potential bias and, thus, enables the more effective analysis of the studies. Recently, under the “umbrella” of EQUATOR (enhancing the quality and transparency of health research), a network was organized and developed for AI reporting guidelines according to study types (i.e., separate guidelines for randomized clinical trials, diagnostic accuracy studies, observational studies, etc.) (Table 2).

## 5. Why Do We Need an AI Reporting Guideline in General?

Research related to algorithm development and the clinical application of AI has also introduced new challenges and obstacles in how such studies are reported, assessed, and compared, in terms of factors that are not specified in traditional reporting guidelines. This could result in missing data and an increased risk of hidden bias. If these actual or potential limitations are not identified, it may lead to implicit approval through publication, which, in turn, may support the premature adoption of new technologies [31]. Conversely, well-designed, well-delivered studies that are poorly reported may be judged unfavorably due to being adjudged to have a high risk of bias, simply due to a lack of information.

The lack of AI clinical study reporting is becoming more widely acknowledged in recent reports. Liu et al. [32] conducted a systematic review including 20,500 articles related to AI. According to independent reviewers who evaluated the confidence in their reported claims, fewer than 1% of these articles were found to be sufficiently robust in their design and reporting. The authors also highlighted the controversy concerning the performance being validated using internal versus external validation, in which internal validation overestimates diagnostic accuracy for both healthcare professionals and deep learning algorithms [32]. In another investigation, only 6% of 516 eligible radiological-AI research publications conducted any form of external validation of their models, and none used multicenter or prospective data collection methods [33]. Similarly, most studies using machine learning (ML) models for medical diagnosis lacked adequate detail on how these were evaluated and they did not provide sufficient information for reproducibility [34]. Inconsistencies have also been reported in how ML models are derived from electronic health records, with details regarding the race and ethnicity of participants omitted in 64% of studies, and only 12% of models being externally validated [35]. Moreover, Nagendran et al. [24] identified high levels of bias in the field, along with a limited availability of datasets and code, which limits the reproducibility of deep learning research to a considerable extent. Descriptions of the hardware used, if present at all, were also brief and this vagueness might affect external validity and re-implementation. All of the above-mentioned concerns arise due to the improper reporting of study design, methodology or algorithms, as well as the fact that most studies do not publicly share the underlying computational scripts in a FAIR manner or provide the underlying CDM data. Taking this together, proper adherence to AI-based reporting guidelines has the potential to minimize possible bias and facilitate reproducibility in research.

## 6. Which AI Reporting Guideline Should I Use for Nephrological Study?

The adherence to a specific AI reporting standard is primarily determined by the primary research or clinical trial task, including whether it is preclinical or clinical, prospective or retrospective, or prognostic or diagnostic, among others [36]. In the last decade, the number of published AI-based clinical studies in medicine, including nephrology, has steadily increased, and the majority of them did not adequately report or comply to the existing AI reporting requirements [24,37,38]. The lack of comprehensive reporting may increase bias and may also have a large influence regarding the reproducibility of the developed model and its final application to the clinical data, along with proper assistance to clinicians for decision making. In each stage of the study, the EQUATOR Network developed specific AI guidelines as an extension of the previous version to standardize AI-based studies, as shown in Table 2. Below, we discuss each developed EQUATOR Network specific AI guideline application based on the study stage.

**Figure 1:**
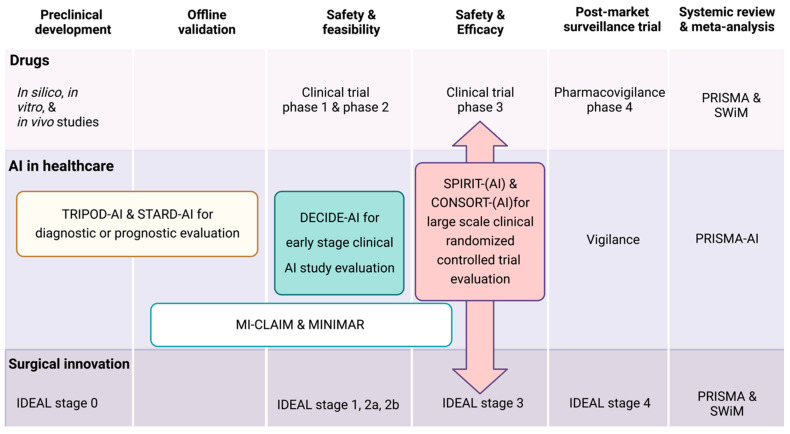
Artificial intelligence reporting guidelines and their application within clinical study stages. The colorful lines reflect reporting guidelines, some of which are specific to research designs (TRIPOD-AI, STARD-AI, SPIRIT/CONSORT, and SPIRIT/CONSORT-AI), while others are stage specific (DECIDE-AI and IDEAL). As a starting point for a broader AI-study application, the MI-CLAIM and MINIMAR standards were utilized. Depending on the circumstances, many research designs may be applicable for each step. **Abbreviations:** AI: Artificial intelligence; CLAIM: Checklist for Artificial Intelligence in Medical Imaging; CONSORT-AI: Consolidated Standards of Reporting Trials–Artificial Intelligence; DECIDE-AI: Developmental and Exploratory Clinical Investigations of Decision support systems driven by Artificial Intelligence; MI-CLAIM: Minimum Information about Clinical Artificial Intelligence Modeling; MINIMAR: Minimum Information for Medical AI Reporting; PRISMA-AI: Preferred Reporting Items for Systematic Reviews and Meta-Analyses–Artificial Intelligence; SPIRIT-AI: Standard Protocol Items: Recommendations for Interventional Trials–Artificial Intelligence; STARD-AI: Standards for Reporting of Diagnostic Accuracy Studies-AI; SWiM: Synthesis Without Meta-analysis; TRIPOD-AI: The Transparent Reporting of a multivariable prediction model of Individual Prognosis Or Diagnosis-AI.

***Diagnostic accuracy study***: The application of diagnostic and prognostic AI algorithms is becoming more popular in nephrology and urology, such as in kidney transplant pathology [39,40,41], delayed graft function prediction [42,43,44], kidney transplant survival [45], and medical image analysis to detect glomerulosclerosis [46,47,48]. Interestingly, AI-provided diagnostic accuracies are similar to those provided by expert clinicians, which might significantly save healthcare resource use [32,49]. Currently, a vast proportion of potential AI/ML-powered healthcare applications are diagnostic AI algorithms; however, the majority of them have been disseminated in the absence of AI-specific reporting guidelines [49]. In terms of study design and data analysis methods, the diagnostic test accuracy studies that are extensively used in nephrology might be reported according to the STARD [50] guideline, if a traditional statistical data analysis method is used. However, for diagnostic studies with AI-intervention, STARD-AI [51] is well suited (Figure 1). Furthermore, besides the comprehensive reporting of research that uses AI algorithms to assess diagnostic test accuracy and performance, STARD-AI may also be used within studies that report on image segmentation and other relevant data classification techniques [49]. The TRIPOD-AI reporting standards may be more applicable, if the emphasis of the study is on establishing, validating, or updating a multivariable prediction model that generates an individualized chance of acquiring a disease (e.g., time-to-event prediction).

***Early stage clinical evaluation (small-scale study) (ESCE):*** ESCE is important for the validation of the performance and safety, similar to phase 1 and phase 2 pharmaceutical trials, prior to phase 3 efficacy evaluation. The best example is the consensus-based reporting guideline for the Developmental and Exploratory Clinical Investigations of Decision support systems driven by Artificial Intelligence (DECIDE-AI) [52]. The guideline is intended to be used in early stage, small-scale clinical studies of AI interventions, when the intervention itself and the human–machine interaction are still refined prior to full evaluation (Figure 1). DECIDE-AI places emphasis on the evaluation study stage and does not prescribe a fixed study design, while STARD-AI [51] and TRIPOD-AI [53,54] are specific to particular study designs. Adherence to these guidelines might be important to prevent a dataset shift, which occurs when an ML-based system underperforms due to an interoperability error or mismatch between the data it was trained on and the data the system encounters after deployment [55,56]. This might cause substantial variation in clinical performance and expose patients to potential unexpected harm.

***Comparative prospective evaluation (randomized controlled clinical trials [RCTs, Phase 3]):*** The SPIRIT (**S**tandard **P**rotocol **I**tems: **R**ecommendations for **I**nterventional **T**rials) and the latest version of the CONSORT (**Con**solidated **S**tandards **O**f **R**eporting **T**rials) statements were published more than a decade ago and provide evidence-based recommendations to improve the completeness of the reporting of randomized controlled clinical trials (RCTs) [57]. While AI systems have been researched for some time, recent advances in deep learning approaches have garnered significant interest for their potential use in healthcare [58]. Consequently, interested parties, experts, and stakeholders have developed the SPIRIT and CONCORT reporting guidelines extensions [59]. These are new reporting guidelines for clinical trial protocols to evaluate interventions, developed in accordance with the EQUATOR Network’s methodological framework, including an AI component [59]. SPIRIT-AI and CONSORT-AI are well-suited for large-scale, randomized controlled clinical trials with AI intervention features (also known as phase 3 for efficacy evaluation) (Figure 1). One of the distinctions between SPIRIT-AI and CONCORD-AI is that the SPIRIT-AI guideline focuses on defining standard protocols for clinical trials, whereas CONSORT is aimed at primary reports of completed randomized trials, with two-group parallel designs. Lately, the SPIRIT group developed reporting guidelines for the molecular and cellular pathology content in clinical trial protocols as an extension [60]. A recent systematic review of RCTs for ML interventions by Plana et al. [25] demonstrated that almost all AI-RCTs follow neither SPIRIT-AI, nor CONCORT-AI, nor any other common AI reporting guidelines. Their initial search yielded 28,159 records and a subsequent, final inclusion resulted in only 41 eligible RCT studies for meta-analysis, indicating a translational gap between development and clinical impact. Among the 41 RCTs that were ultimately included in the analysis, none of them fully adhered to all CONSORT-AI standards. Common reasons for non-adherence included not assessing poor-quality or unavailable input data (38 out of 41 trials (93%)), not analyzing performance errors (38 out of 41 (93%)), not including a statement regarding code or algorithm availability (37 out of 41 (90%)), and enrolling only a small number of participants from underrepresented minority groups [25]. This may indicate that many FDA-approved, ML-enabled medical devices, which are approved with only a limited amount of clinical data FAIRification during an RCT [25]. To sum up, the quality of medical ML-centric RCTs, as well as their underlying reporting transparency and inclusion, should be carefully addressed by adhering to one of the existing AI reporting guidelines when designing or publishing future trials.

***Clinical image analysis:*** The **C**heck**l**ist for **A**rtificial **I**ntelligence in **M**edical Imaging (CLAIM) was developed in 2020 to assist scientists presenting research and to analyze previously published AI applications in medical imaging [61]. The CLAIM checklist, which was inspired by the Standards for Reporting of Diagnostic Accuracy Studies standards [50], was created specifically to address AI applications in medical imaging, including classification, detection, reconstruction, and workflow optimization, among others [61]. The CLAIM checklist includes 42 criteria for presenting medical imaging AI research that should be regarded or viewed as best practice. Recently, Belue et al. demonstrated a low rate of adherence to the CLAIM reporting guideline among published prostate MRI applications [62]. Here, the authors analyzed 53 studies and most of them did not follow the CLAIM checklists. Among the unreported items from a total of 42 items contain de-identification methods, as follows: item 13 (68% no): handling missing data; item 15 (47% no): rationale for choosing ground truth reference standard; item 18 (55% no): measurements of inter- and intrareader variability; item 31 (60% no): inclusion of validated interpretability maps; and item 37 (92% no): inclusion of failure analysis to elucidate AI model weaknesses. Moreover, an area under the curve (AUC) analysis of the CLAIM fulfillment quartile revealed a significant difference of the mean AUC scores between quartile 1 versus quartile 2 (*p* < 0.034) and quartile 1 versus quartile 4 (*p* < 0.003) scores [62]. This result may suggest that a higher adherence to the CLAIM may improve AI model performance.

***Systematic review or meta-analysis:*** Systematic reviews serve a variety of important purposes. They can provide summaries of the state-of-the-art in a field, allowing future research priorities to be identified; they can answer questions that individual studies would be unable to answer; they can identify problems in primary research that should be addressed in future studies; and, finally, they can generate or evaluate theories about how or why phenomena occur. The initial **P**referred **R**eporting **I**tems for **S**ystematic **R**eviews and **M**eta-**A**nalyses (PRISMA) statement was published in 2009 and the latest update was made in 2020 to assist systematic reviewers in reporting why the review was conducted, what the authors conducted, and what was discovered [63]. The continuous increase in AI-related studies in medicine required an AI-extension of the PRISMA guideline to standardize AI-based systematic review and meta-analysis reports and interpretations. The development of the PRISMA-AI extension focuses on standardizing the reporting of methods and results for clinical studies using AI, reflecting the most relevant technical details required for future replicability, as well as the clinician’s ability to critically follow and ascertain the relevant outcomes of such studies [64]. In some cases, when conducting systematic reviews examining the quantitative effects of interventions, for which meta-analysis of effect estimates is not possible or not appropriate for a least some outcomes, the **S**ynthesis **Wi**thout **M**eta-analysis (SWiM) reporting guideline can be utilized [65].

## 7. What Are the Minimal Requirements for AI Reporting Guidelines?

The American Medical Informatics Association published the MINIMAR (**Min**imum **I**nformation for **M**edical **A**I **R**eporting) guideline in June 2020, which is not part of the EQUATOR reporting guidelines [66]. The guidelines are intended for studies that describe the use of AI systems in healthcare. Their goal is to ensure that the minimum amount of information required to adequately understand an AI algorithm’s intended predictions, target populations, and potential biases is reported clearly and comprehensively. Unlike other reporting guidelines, which provide a checklist of items that must be reported by researchers, these guidelines offer recommendations for reporting information in four primary areas of clinical AI studies, as follows: (1) study population and setting; (2) patient demographic information; (3) model architecture; and (4) transparently reporting model evaluation, optimization, and validation to clarify how local model optimization can be achieved while also allowing replication and resource sharing. There is an overlap between the MINIMAR [66] guidelines and the minimum information about clinical artificial intelligence modeling (MI-CLAIM) [67] guidelines, which both focus on AI algorithms and how they were developed and validated with regards to reproducibility via the FAIR principles. Both MINIMAR and MI-CLAIM emphasize the minimal essential information that should be disclosed in an article. The MI-CLAIM comprises six sections. In section one, it is essential to describe the study as a whole and can be broken down into four subsections, as follows: (a) clinical setting, (b) performance measures, (c) population composition, and (d) current baselines to measure performance against. In section two, it is essential to partition model training and model testing [67]. In section three, it is clearly specified how the data were cleaned and formatted, and, if relevant, what data were additionally available but not used (also known as model optimization and selection). Performance evaluation (F scores, Dice coefficient, or area under the curve (AUC)) is section four in the MI-CLAIM. This section will include typical results showing the performance of the baseline and new models tested, as well as appropriate statistics for significance. The results of the model examination have to be evaluated in light of the model’s performance, indicating that the results of examining a model with excellent performance metrics for a specific clinical task should be regarded as more relevant than the results of examining a lower-performing model for the same task [67]. The final section is reproducibility. The goal here is not for an independent researcher to reproduce the exact results, but rather to replicate the exact process by which the results were generated, giving that second investigator everything they need to rapidly validate the results in their own cohorts [67].

## 8. What Else Could Be Done for an Improved Guideline Adherence and the Use of AI Models in Nephrology?

To allow for a more versatile use of AI approaches in clinics, high-quality AI models need to be developed for the particular end user, i.e., clinicians. This still points to the currently published challenges for nephrology [68] that need to be addressed, in addition to the already mentioned concepts of FAIR and the use of CDMs—data availability and usability.

1. *Synthetic data as a digital twin of real-world patient data:* Synthetic data is computer-generated data that mimics real-world data, while preserving its statistical properties [69,70]. Thus, it can enable researchers to share and collaborate on nephrology-related studies without risking the exposure of sensitive patient information. By sharing synthetic data, researchers can access larger and more diverse datasets, leading to more robust and generalizable findings. This approach fosters collaboration between institutions and researchers, accelerating advancements in the understanding, diagnosis, and treatment of kidney diseases, among others, while maintaining patient privacy and adhering to regulatory requirements [70].
2. *Predictive modeling:* Synthetic data can be used to create large, diverse datasets that help to develop predictive models for various kidney diseases. These models can assist clinicians in predicting which patients are at high risk for developing kidney disease or experiencing complications. In addition, this can enable researchers to identify patterns and trends that may not be evident in smaller, less diverse datasets.
3. *Development of software requiring patient data:* Synthetic data can also be used to develop software that requires patient data, like clinical decision support systems that assist clinicians in making treatment decisions for patients with specific kidney diseases. For instance, a decision support system could utilize synthetic data for training purposes to recommend the best treatment options for patients based on their clinical characteristics.

**Usability and technology acceptance to ultimately bring solutions for clinicians into their daily routine:** For clinicians, both usability and technology acceptance are critical factors to consider when evaluating and implementing new clinical procedures [71]. A technology that is easy to use and fits seamlessly into their workflow is more likely to be adopted and used effectively [72].

1. *User-centered design:* The design of the AI system should be centered around the needs of the clinicians who will be using it. The system should be intuitive and easy to use, with a user interface that is easy to navigate. For example, AI-based support systems can be used to develop and implement clinical decision rules in nephrology [73]. These decision rules can support clinicians to obtain more timely decisions, such as when to initiate dialysis or refer a patient for a kidney transplant.
2. *Integration with clinical workflow:* The AI system should be integrated into the clinical workflow in a way that minimizes disruption and maximizes efficiency [74]. This may involve integrating the system into existing EHR systems or other clinical tools already in place. In addition, diagnostic procedures in nephrology would depend on the ability to integrate data from various sources beyond EHR, such as laboratory test results, imaging data, or clinical trials. For example, these systems can predict the risk of related developing complications, such as the risk of progressing kidney failure [74].
3. *Training and education:* Clinicians need to be trained on how to use the AI system effectively [75]. This may involve providing training on the system itself, as well as on the underlying data and algorithms, because clinicians need to understand how the AI system works and how it arrives at its recommendations. The system should be transparent and provide clear explanations of its recommendations, so that clinicians can make informed decisions.
4. *Healthcare regulators workplan:* Aligned with the FDA’s enduring dedication to create and employ innovative strategies for overseeing medical device software and other digital health technologies, in April of 2019, the FDA released the “Proposed Regulatory Framework for Modifications to Artificial Intelligence/Machine Learning (AI/ML)-Based Software as a Medical Device (SaMD)-Discussion Paper and Request for Feedback”. This document outlined the FDA’s groundwork for a potential method of premarket evaluation for modifications to software driven by artificial intelligence and machine learning. However, the current challenges and rapid developments in the AI healthcare industry need more aggressive action from authorities to put them on one stream. Recently, the European Medicines Agency (EMA) and the Heads of Medicines Agencies (HMAs) have released a comprehensive artificial intelligence (AI) roadmap through 2028, outlining a united and synchronized approach to optimize the advantages of AI for stakeholders, while mitigating the associated risks. Here, the Common European data spaces are a key initiative aimed at unleashing the vast potential of data-driven innovation in the EU. They will facilitate the secure and trustworthy exchange of data across the EU, allowing businesses, public administrations, education, and individuals to maintain control over their own data while benefiting from a safe framework for sharing it for innovative purposes [76]. This initiative is crucial for enhancing the development of new data-driven products and services, thereby potentially forming an integral part of a connected and competitive European data economy. Complementing these data spaces, the European Commission is also addressing the risks associated with specific AI uses through a set of complementary, proportionate, and flexible rules, aiming to establish Europe as a global leader in setting AI standards. This legal framework for AI, known as the AI Act, brings clarity to AI developers, deployers, and users by focusing on areas not covered by existing national and EU legislations [77]. It categorizes AI risks into four levels, as follows: minimal, high, unacceptable, and specific transparency risks; it introduces dedicated rules for general purpose AI models. Together, these measures may represent a comprehensive approach to foster a safer, more trustworthy, and innovative data and AI landscape in Europe. However, the current challenges and rapid developments in the AI healthcare industry need more aggressive action from authority organizations such as the FDA, EMA, and PMDA to develop unified regulatory guidelines.

## 9. A Perspective of Generative Language Processing Utilization in Nephrology

The field of nephrology is increasingly using advanced technologies to improve patient care, diagnosis, and research. Generative AI language processing, a subset of natural language processing (NLP), aimed at creating human-like text, has emerged as a useful instrument in this endeavor. Generative language processing, utilizing machine learning and linguistic analysis, provides novel solutions to medicine challenges such as data analysis, clinical documentation, patient communication, and medical education [78].

*Generative AI in clinical documentation:* One of the primary applications of generative language processing in medicine, including nephrology, is to improve clinical documentation. Electronic health records (EHRs) store huge amounts of unstructured data, such as clinician notes, laboratory results, and imaging reports. Extracting useful data from these records can be time-consuming and error prone. Generative language models trained on medical text can automate the summarization and extraction of key clinical information, allowing nephrologists to document their cases more efficiently [78].

*Improving Diagnostics and Patient Management.* In the near future, generative language processing might improve patient care and diagnostics in medicine, including nephrology. By analyzing patient data such as laboratory values, vital signs, and clinical notes, machine learning algorithms can help clinicians identify patterns and predict results. For example, generative models can assist nephrologists in identifying patients at high risk for acute kidney injury or the progression of chronic kidney disease, allowing for an early intervention and personalized treatment strategies.

Despite its potential, using generative language processing in nephrology presents some challenges. Data privacy, algorithm bias, and clinical validation are all issues that must be carefully addressed to ensure that these technologies are used carefully and ethically. Furthermore, additional research is required to optimize generative models for specific nephrology applications and assess their impact on patient outcomes and healthcare delivery. In near perspectives, generative language processing has the potential to significantly improve patient care, research, and education in nephrology. Clinicians and researchers can use artificial intelligence and natural language understanding to gain new insights, streamline workflows, and improve the quality of care for patients with kidney diseases.

## 10. Conclusions and Future Perspectives

This publication aims to assist researchers across various medical specialties, especially in nephrology, in better understanding, selecting, and implementing AI reporting criteria for their studies. Finally, the impact of AI-specific reporting guidelines, along with the related upstream processes, such as CDMs and the FAIR principles, on improving the quality of AI healthcare research largely depends on the extent to which researchers utilize them when reporting studies; medical journal editors require authors to employ them when submitting studies and reviewers apply them when appraising studies. As demonstrated in this study, the number of AI-powered clinical research studies in nephrology is steadily increasing; nevertheless, adherence to AI-specific standards and usability aspects can lead to improved adoption by clinicians, aiding them in clinical decision making.

## Figures and Tables

**Table 1:** Key Box.

Key Box
**Artificial intelligence:** Artificial intelligence (AI) is a general term that implies the use of a computer to model intelligent behavior with minimal human intervention. **Machine learning:** Machine learning is one of the branches of artificial intelligence (AI), which focuses on the use of data and algorithms to imitate the way that humans learn, gradually improving its accuracy. **Deep learning:** Deep Learning is one type of machine learning algorithm that uses artificial neural networks that can learn extremely complex relationships between features and labels and have been shown to exceed human abilities in performing complex tasks [7]. **Ground truth:** This refers to the correct or “true” answer to a specific problem or question. In the biomedical field, it is a “gold standard” guideline, expert opinion, or clinically proven outcome that can be used to compare and evaluate model results. **Black box algorithms:** These are not used to explain or justify obtained results, i.e., neural network-trained and identified outcomes are mostly hard to explain even with a high accuracy prediction [8].

**Table 2:** AI reporting guidelines.

Name	Stage of Study	Application in Nephrology or Other Healthcare Fields	EQUATOR Reporting Guidelines
TRIPOD-AI	Pre and clinical development	Extension of TRIPOD guideline used to report prediction models’ (diagnostic or prognostic) development, validation, and updates.	Yes
STARD-AI	Pre and clinical development	Extension of STARD guideline used to report diagnostic test accuracy studies or prediction model evaluation.	Yes
DECIDE-AI	Early clinical study stage evaluation	Used to report the early evaluation of AI systems as an intervention in live clinical settings (small-scale, formative evaluation), independently of the study design and AI system modality (diagnostic, prognostic, and/or therapeutic).	Yes
SPIRIT-AI	Comparative prospective evaluation	Extension of SPIRIT guideline and mainly uses randomized trials.	Yes
CONSORT-AI	Comparative prospective evaluation	Extension of CONSORT guideline and mainly uses clinical trial protocols.	Yes
PRISMA-AI	Systemic review analysis	Extension of PRISMA guideline, which are used for meta-analysis or systemic review analysis.	Yes
CLAIM	Medical image analysis	Extension of the STARD reporting guideline. CLAIM is used in AI medical imaging evaluations that include classification, image reconstruction, text analysis, and workflow optimization. The majority of autosomal dominant polycystic kidney disease and renal cancer CT or MRI images are used, but AI analysis studies did not adhere to the CLAIM guidelines.	Yes
MI-CLAIM	Minimal clinical AI modeling research	The guidelines are designed to inform readers and users about how the AI algorithm was developed, validated, and comprehensively reported. They are split into six parts: (1) study design; (2) separation of data into partitions for model training and model testing; (3) optimization and final model selection; (4) performance evaluation; (5) model examination; and (6) reproducible pipeline.	Yes
MINIMAR	Minimal healthcare AI modeling studies	MINIMAR reporting guideline stand upon four essential components: (1) study population and setting; (2) patient demographics; (3) model architecture; and (4) model evaluation. This reporting guideline can be applied for almost all healthcare studies.	No

**Abbreviations:** CLAIM: Checklist for Artificial Intelligence in Medical Imaging; CONSORT-AI: Consolidated Standards of Reporting Trials–Artificial Intelligence; DECIDE-AI: Developmental and Exploratory Clinical Investigations of Decision support systems driven by Artificial Intelligence; MI-CLAIM: Minimum Information about Clinical Artificial Intelligence Modeling; MINIMAR: Minimum Information for Medical AI Reporting; PRISMA-AI: Preferred Reporting Items for Systematic Reviews and Meta-Analyses–Artificial Intelligence; SPIRIT-AI: Standard Protocol Items: Recommendations for Interventional Trials–Artificial Intelligence; STARD-AI: Standards for Reporting of Diagnostic Accuracy Studies-AI; TRIPOD-AI: The Transparent Reporting of a multivariable prediction model of Individual Prognosis Or Diagnosis-AI.
